# Topics of Public Interest

**Published:** 1912-05

**Authors:** 


### TOPICS OF PUBLIC INTEREST

### Standardization of Disinfectants

J. T. Ainslee Walker, F. R. S. M., F. C. S., London; now located at the Whitehall Building, New York City, has asked our cooperation in a movement to secure the standardization of disinfectants. Maryland is the only state thus far exercising control over the sale of such substances.

(EXTRACT from State Board of Health of Maryland, Regulation No. 2—Labeling of Disinfectants. Revised to May 1, 1911.)—All disinfectants manufactured or sold in this State must bear a label showing the carbolic acid coefficient, or relative germicidal strength of such disinfectants as compared with pure carbolic acid.

In determining the relative germicidal value of disinfectants the application of the Rideal-Walker Test to the typhoid bacillus in a 24 hour bouillon culture may be made, and such results will be accepted until further notice.

The statement of the coefficient should be made as follows:

Carbolic Acid Coefficient 0.3, or 1.2 etc., etc.

This statement may appear on the principal label or on a supplemental label or sticker.

---

### Warren Triennial Prize—Massachusetts General Hospital

THE subject for competition for the year 1913 is on SOME SPECIAL SUBJECT IN PHYSIOLOGY, SURGERY OR PATHOLOGY.

Dissertation must be in English, French or German, typewritten and bound, so as to be easily handled. The name of the writer must be enclosed in a sealed envelope, on which must be written a motto corresponding with one on the accompanying dissertation. The amount of the prize for the year 1913 will be $500. In case no dissertation is considered sufficiently meritorious, no award will be made. Dissertations will be received until April 14, 1913

FREDERIC A. WASHBURN,
*Resident Physician.*

---

### Vaccination Statistics

DURING the first three months of 1912, there were reported 283 cases of smallpox in Michigan. The vaccination history of these cases is as follows:

2 cases vaccinated, "50 or 60 years ago"; 3 cases vaccinated, "14 years ago"; 1 case vaccinated, "years ago"; 1 case vaccinated, "at the time of exposure"; 1 case vaccinated, "12 years ago"; 1 case vaccinated, "infancy and again 10 years ago"; 1 case vaccinated, "about 10 years ago"; 1 case vaccinated, "some 20 years ago"; 1 case vaccinated, "one week after exposure"; 10 cases vaccinated, "about 5 years ago" (some doubt); 1 case vaccinated, "some years previous"; 2 cases vaccinated, "in childhood"; 2 cases vaccinated, "when very young"; I case vaccinated, "30 years ago"; 8 cases vaccinated, "6 years ago"; 1 case vaccin- "2 years ago"; 1 case vaccinated, "4 years ago"; 1 case vaccinated, "5 years ago"; 5 cases vaccinated, "doubtful if ever"; 245 cases, "NEVER VACCINATED"; total, 283.

It costs Michigan $150,000 a year to take care of indigent smallpox patients and to protect the unvaccinated.

R. L. DIXON, Secretary,
Michigan State Board of Health.

---

### Oneida County Provides Hospital Care for All Citizens, Whether Paupers or Self-Supporting

UTICA, April 14th—Governor Dix has signed a bill extending to all of the sick in Oneida County the same opportunity for hospital care as is now provided for those sick with tuberculosis by the county tuberculosis hospital law.

Like many other countries, Oneida has a hospital connected with its almshouse. Recently the county built a large hospital and has decided to separate its management and control entirely from that of the almshouse. The county authorities found many sick persons in the county, especially in the rural sections, who should have hospital care. They are unable to get it in city hospitals and refuse to go to the county hospital if it is necessary to suffer the stigma of pauperism.

Under the new law the hospital will be open to all citizens of the county, whether they are able to pay for their care or not. This is in line with movements in California and other States which provide county hospitals about as they provide public schools.

The control of the hospital will be vested in a Board of Managers consisting of five citizens, two of whom shall be physicians who serve without pay.

There is just one thought that occurs to us in connection with the above: The Poor House, County House, or County Hospital, is about the one method of "pauperization" which ought to be free from the stigma. It does not depend on voluntary charity but is supported by equable public taxation and is maintained on an economic basis. The dependent person in need of care, who accepts this form of charity is indebted only to the public. If he has ever paid taxes, even indirectly, we can see no more reason for feeling that he is pauperized, than if he accepts insurance after having paid the premiums. If he has not been a tax payer, he is pauperized in no truer sense, though perhaps to a greater financial amount, than if he drew a book out of a public library, enjoyed a park provided by the city or state. The person who accepts private charity, asks for special privileges and for support on a higher scale of expense, in a hospital not supported by the public in its official capacity, is really the one that is pauperized.

### Universal Peace.

Dr. J. A. Riviere, of Paris, Chevalier of the Legion of Honor, and Editor-in-chief of the Annals of Physico-therapy, sends us a circular addressed to the members of the International Medical Association against War. Some of the ideas expressed are more progressive and elaborate than the usual pleas for peace as an ethical and economic desideratum. Among these are: the inviolability of the principle of supply and demand; that neither force of numbers nor that of wealth, should overcome the work of the time regulated by free meeting and exchange of thought; that idleness is a crime against society; that the state should study to utilize wasted energy; that dirigibles and aeroplanes should not be used in war; that writings, shows and moving pictures should be used for popular education and uplift; that certain sports which endanger health and life should be regulated.

---

### Chiropodists placed under State Board Regulation.

Since 1895, there has been some sort of regulation of chiropody, mainly under the direction of a board elected by the "Pedic Society." There has gradually developed a sentiment for stricter and more impartial regulation and the Chiropodists of America, through their Secretary, have issued a letter from which we quote the following:

The new law just enacted dissolves the Pedic board of examiners and in effect provides that on and after September 1, 1912, no person not heretofore legally authorized to practice shall be permitted to engage in such practice until he shall have been duly licensed so to do by the Regents of the University of the State of New York and on the recommendation of the State Board of Medical Examiners. It then provides in detail for the examination and the qualifications of the applicants and of schools intending to teach chiropody and also for the issuance of licenses and the revocation of same, all under the sole supervision of the Board of Regents in connection with the State Medical Board and it gives the Regents plenary power to work with.

The bill was drawn after consultation with the Education Department and has its approval and also the approval of the State Medical Board.

The Pedic Society of the State of New York fully indorsed this bill, believing that the best interests of all concerned will be conserved thereby. The registered chiropodists of this State in no wise conflict with the medical profession, as physicians consider it infra dig. to practice chiropody, and in most instances send persons having corns, warts, callosities, ingrowing nails and bunions to chiropodists. In these instances proficient services should in all respects be forthcoming, and there is no better way of perfecting the services than by placing the examining power and all the authority in this matter in the hands of the Regents and the State Medical Board.

New Jersey has a law similar to the proposed law which provides that the examinations be in the hands of the Board of State Medical Examiners. As there is no Board of Regents in New Jersey, there is no provision for such a Board in the New Jersey chiropody law. The same has worked very satisfactorily and a number of other States have enacted or are about to enact similar laws.

---

### Tuberculosis.

THERE has been a controversy between Dr. Charles S. Prest of the State Health Department and Dr. George Washington Goler, Health Commissioner of Rochester, as to the law regarding disinfection after tuberculosis. Mr. Homer Folks states that the law does not compel physicians to disinfect, thus supporting Dr. Goler.

Lockport has secured a visiting nurse for tuberculosis cases—Miss Mary G. O'Donnell, late of Bay View Hospital, Baltimore. At the beginning, her salary will be paid by funds raised from the sale of red cross stamps and private contributions. Later, it is hoped that the city will support the work. Commencing her duties on April 4, she visited 23 victims in the next two weeks and found a number of cases not previously known to the health board.

Tonawanda and North Tonawanda have voted $175 apiece for expenses of the Tuberculosis exhibit to be held in the armory on Delaware Street, May 5 to 10, under the direction of the State Board of Health.

---

NEW Jersey has just passed a stringent anti-tuberculosis law. Every tuberculosis case refusing to obey regulations of the State Board of Health, shall be compulsorily seggregated in special institutions which the counties must provide by Oct. 1, 1912. If the patient's family is unable to pay, the State will reimburse the County at the rate of $3.00 weekly. Maryland has a similar law but less drastic. San Francisco is the only city in the country which has a similar ordinance, but New York and several other cities exercise the same power by construction of more general ordinances.

The National Association for the Study and Prevention of Tuberculosis, while approving these laws, holds that "a consumptive who exercises sufficient precautions in the disposal of his sputum need not be a menace to anyone." With this, highly approved dictum, we venture to disagree. If all tubercle bacilli were contained in sputum voluntarily spit out, the dictum would be correct. But, how about faeces, urine, other discharges? Healthy persons sneeze, cough, drool at night, use their handkerchiefs, moisten their lips, and make various reflex or unguarded voluntary movements which bring fingers thus moistened, in contact with hair, clothing, towels, etc. We all use from 25 to 50 different table utensils at a meal. There is also, in the terminal stage of tuberculosis, a period of days or sometimes even months, in which no one can be careful or conscientious, being either unconscious or in such a state of hebetude as to be irresponsible. With abundant faciliites for the prompt disinfection of clothing, linen, dishes, etc., and with rooms built so as to be readily sterilized, the contamination may be counteracted. In a house or flat of ordinary construction, with many cracks, with porous floors, wall, and furnishings, with practically no part either air tight or water tight and many parts or whole rooms almost sun-tight, we fail to see how contamination can fail to become considerable, as days lengthen to weeks and months, and the thousand to one chances against infection are multiplied by a million. And how can disinfection be practiced, so as to be at the same time efficient and economically possible?

---

### Water Supplies.

MIDDLEPORT will soon install a water supply at a cost of about $50,000 and sewerage at a cost of $35,000. The plans and specifications have been approved at Albany and bids are being considered. There will be a pumping station, stand pipe and about ten miles of water mains.

Corning faces the necessity of filtering her present water supply or seeking a different source. Dr. F. S. Swain, the Health Officer, Dr. E. H. Hutton and Mr. W. T. Moran have been appointed a committee to investigate. An epidemic of typhoid is in progress.

The Niagara Frontier Pure Water Conference representing especially, Buffalo, the Tonawandas, Lockport and Niagara Falls, will hold a dinner May 10, 1912, in the Armory at Tonawanda. Dr. Eugene H. Porter, State Health Commissioner will speak and it is hoped that the Secretary of War, Henry L. Stimson, will attend. The object of this conference is not merely to secure local improvements but to further international legislation to abolish contamination of the entire Great Lakes system, including tributaries. Congressman James S. Simmons of Niagara

Falls, has already introduced a bill with this object in view, and this bill is favored by the conference and, indeed, may be passed at the present session of congress.

---

MAYOR KELLER, of Niagara Falls, after consulting the members of the water commission, has asked State Health Commissioner Eugene H. Porter, to close the private water works till their filter plant is completed or to get a supply of wholesome (?) water from municipal mains.

---

THE STATE INSTITUTE FOR THE INVESTIGATION OF MALIGNANT DISEASE, has received bids for the construction of a cancer hospital, to be conducted in connection with the Gratwick Laboratory. The lowest bidder was the Eastern Concrete Steel Co., $65,219. The highest bid was for $77,113. All bids were by Buffalo firms.

---

LONG Hat Pins are now forbidden by law in New Orleans. Freak legislation, isn't it—until you have known of case when a pin did penetrate an eye?

---

ECONOMY IN ADMINISTRATION OF THE HEALTH DEPARTMENT OF BATAVIA. Last year, a la carte examination of cultures for diphtheria, cost $6,000, owing to the prevalence of this disease. This year, the Board of Health has fixed the salary of the Health Officer at $600, with an allowance of $100 for expenses. This is an American plan rate, for taking and examining cultures, fumigating, etc. As a taxpayer, we are heartily in favor of economy and we believe that, when the medical work of a governmental unit, or even of an institution reaches sufficient bulk, it is perfectly proper to contract for the services of a physician or pathologist or other expert, instead of paying the retail rates equally just and proper for an occasional service. But, we question seriously whether there is to be expected any such fluctuation in amount of work as the figures indicate and we believe, that, whatever system is employed, a fair rate should be insisted upon.

---

DUNKIRK has instituted a municipal laboratory for the examination of water, milk, etc., and for making "blood tests," whatever that may imply. Dr. C. E. Hollenbeck has been engaged as director at a salary of $25 a month. Thus is the medical profession waxing rich at the expense of the poor taxpayers. Can nothing be done to deplete such plethora?

The Legal Aid Bureau of Buffalo, with offices at 448 Ellicott Square, has been established for a purpose clearly indicated by its title. Medical dispensaries, free to the poor have been operated for several centuries and the question has often been asked why there were not similar legal dispensaries. To a certain extent, the assignment of a lawyer by the court to defend a poor man under criminal charge, the method—not generally favored—of conducting civil cases on a percentage basis, and the solicitation of legal aid by private individuals and philanthropic institutions, of lawyers, for definite cases, have filled the vacancy but there is no question but that this Bureau will fill a long felt need. There is only one thing about the prospectus that we do not quite like—the solicitation of financial aid from the medical profession, which is already conducting a precisely analogous charity on a far larger scale and which has more than enough other demands on his meagre earnings.

---

The State Department of Health will hold Institutes for the instruction of local health officers, in Chemung, Steuben, Tioga, Schuyler, Yates, Livingston, Broome, Allegany, Cortland and Tompkins Counties, during the next few months.

---

On Wednesday, April 17, the German Deaconess Hospital graduated a class of eight nurses. Two of them, by the way, have German names.

---

A fire occurred April 20, in the locker-room of the University of Buffalo. Dr. James A. Gibson, superintendent of the building, discovered the fire, and with the help of several students, extinguished it without turning in an alarm.

The damage amounts to about $50.00.

---

### Cost of Food.

Mayor Shank of Indianapolis addressed the Housewives' League of Buffalo, April 19, detailing some of his work as voluntary purchasing agent and showing the relatively enormous cutting of middle men's profits possible. It is a sign of awakening common sense that people are no longer afraid of being called "tight-wads" for exercising the same supervision over food prices as those of clothes, rent, heating, etc.

Recently, we had had occasion to glance through several account books, dating back to 1855 in Buffalo, to about 1840 in Albany, even then regarded as an expensive city in which to live, and, in the country to about 1790. With many exceptions and with due allowance for differences in local conditions and scale of living, it has impressed us that the principal general increases have been in food stuffs, in labor directly employed, and in luxuries. Rent, for example, has diminished rather than increased, if we allow for the difference in plumbing, maintenance and the fact that taxes, largely derived from landlords, cover enormous expenditures for education, philanthropy and general enjoyment and welfare.

However, the Bureau of Commerce and Labor, presents some statistics showing that we are increasing our imports of food stuffs. In so far as the import are of supplies not grown at home—tea, coffee, cocoa, etc.—the matter is serious only in indicating an increased use of mild stimulants and the constant increase of price. Allowing for difference in population, the importation of alcoholics has shown little increase in 10 years. But, when in February, we imported 2½ million dollars' worth of potatoes, as much as the average importation for a year, we begin to realize that we are not so literally self-supporting as we should be. The importation of fruits and nuts, perhaps indicates a wholesome dietetic tendency and the falling off in imports of raisins and oranges shows that our home production is increasing. But in going outside to buy a million and a half dollars' worth of wheat and flour, nearly six million dollars' worth of cheese, and many more million dollars' worth of goods that are merely dietetic equivalents of home products, in eight months, we acknowledge an inherent weakness.

---

### Duty of Layman to Report Contagious Disease.

FROM Feb. 1 to 26, the little daughter of Mr. W. E. Robertson, a prominent Buffalo business man was out of school and was said to have had Christian Science treatment. Investigation by the health department resulted in a diagnosis of scarlet fever and suit was brought against the father for failure to report the case. On April 11, a verdict of no cause of action was brought in, although the Health Department, through the District Attorney will endeavor to reopen the case. This is the first time that a layman has been on trial under the ordinance requiring contagious diseases to be reported.

After reviewing the legal aspects of the passage of the ordinance and declaring that it is a perfectly sound one from the legal viewpoint, Judge Hager said: "You are not to pass on the reasonableness or unreasonableness of the ordinance; that is a matter for the court. The issue in this case is this: Did the daughter of this defendant have scarlet fever or did she not? If you decide that she did have scarlet fever, then you must decide whether or not this defendant acted in good faith in not reporting the case to the department of health."

Council for the defendant plead that the matter of Christian Science attendance had nothing to do with the case, and laid stress on the question of good faith of the defendant and reasonableness of the ordinance, pointing out the errors in diagnosis made by physicians, and even by the Health Department itself in placing together in the Contagious Hospital, well developed and merely suspected cases. The District Attorney, on the other hand asked the judge to charge the jury that, if he did not know that the child had scarlet fever and failed to report the case which proved actually to be one of scarlet fever, that he was guilty of violating the ordinance. This, as implied above, the judge refused to do and, in fact, charged in an exactly contrary manner.

It is extremely difficult to make satisfactory comment on this case. Naturally, we do not believe in Christian Science but we do believe very thoroughly in what used to be an American policy of individual liberty, provided that the execution of the policy does not endanger others. However, we need not enter into this discussion except as it bears on the question of the duty of a parent or guardian to provide legally qualified medical attendance for a child. Here again, it is obviously difficult to frame a satisfactory law so as to steer between the Scylla of neglect and the Charybdis of calling the doctor for every headache or hysteric tendency. In the present case, the child recovered and, apparently, was not seriously sick.

It is also obvious that, without working injustice to perfectly innocent offenders and requiring a diagnostic acumen that even our profession does not possess, some allowance must be made in the application of any law on this subject, if indeed the working of a law according to the strictest ideas, would not in itself be contrary to the spirit and precedent of law in general.

We have a slight business acquaintance with Mr. Robertson, sufficient to express entire confidenec in his intention to do his duty as a father and a citizen. It is unfortunate that this case establishes a precedent which may be used to subvert the ordinance even in plain cases of serious contagious disease, with neglect to provide medical attendance.

---

DR. R. M. PEARCE, Professor of Research Medicine at the University of Pennsylvania, will deliver at the Syracuse Medical School the annual Alpha Omega Alpha address of the Gamma of New York Chapter on May 21. The title of the address is "Medical Education." The address will be delivered at the annual public meeting of the chapter at 8.30 P. M. in the Hiawatha Room of the Onondaga. At 6.30 P. M. Dr. Pearce will be the guest of honor of members of the fraternity at their annual dinner at the Onondaga.

---

**Medical Interne. Government Hospital for thc Insane.**

EXAMINATIONS will be held June 5, 1912, at various places, for eligibles. At least two vacancies are to be filled. The positions are for a year, paying $50.00 a month and maintenance, with opportunities for promotion if desired. Applications are limited to men graduated not more than two years, unless they have been continuously engaged in hospital or laboratory work. Applications should be made promptly to the U. S. Civil Service Commission, Washington, for blank No. 1312.

---

**New Inventions.**

1,022,459. Fresh-Air Sleeping Hood. Albert Bennett, Seattle, Washington.

1,022,507. Baby-Holder Attachment for Cribs, etc. Marion J. Ross, Boston, Massachusetts.

1,022,601. Vaginal Syringe. Sven S. Rumberg, Batavia, and Felix C. Hartlung, Chicago, Illinois.

1,022,606. Microscope. Karl M. Stahl, New York, N. Y.

1,022,627. Medicinal Bath. Albert Hempel, Leipsig-Oetzsch, Germany.

1,022,645. Pharmaceutical Product. Ludwig Taub and Hans Hahl, Elberfield, Germany. Assignors to Friedr. Bayer & Co., Elberfield, Germany.

1,022,894. Catamenial Napkin. Elizabeth Sprague, Minneapolis, Minnesota.

1,022,443. Bottle Stopper, Maurice W. Send, Detroit, Michigan.

1,022,444. 2-Phenylquinolin 4-Carboxylic-Acid Ester Ludwig Taub, Elberfeld, Germany.

1,022,491. Fumigator, Peter McCarthy Judson and Edward Michmershurzen, Holland, Michigan.

1,022,548. Drinking Cup, August H. Hartman, Seattle, Washington.

1,023,042. Syringe, Allen E. Scott, San Francisco, Cal.

